# FOXP3 promotes tumor growth and metastasis by activating Wnt/β-catenin signaling pathway and EMT in non-small cell lung cancer

**DOI:** 10.1186/s12943-017-0700-1

**Published:** 2017-07-17

**Authors:** Shucai Yang, Yi Liu, Ming-Yue Li, Calvin S. H. Ng, Sheng-li Yang, Shanshan Wang, Chang Zou, Yujuan Dong, Jing Du, Xiang Long, Li-Zhong Liu, Innes Y. P. Wan, Tony Mok, Malcolm J. Underwood, George G. Chen

**Affiliations:** 1 Department of Clinical Laboratory, Pingshan District People’s Hospital Of Shenzhen, Shenzhen, China; 2 Department of Surgery, the Chinese University of Hong Kong, Prince of Wales Hospital, Shatin, NT Hong Kong, China; 3 Guangdong Key Laboratory for Research and Development of Natural Drugs, Guangdong Medical University, Zhanjiang, Guangdong China; 4 Department of Clinical Oncology, the Chinese University of Hong Kong, Prince of Wales Hospital, Shatin, NT Hong Kong, China; 5 Department of Otorhinolaryngology, Head and Neck Surgery, the Chinese University of Hong Kong, Prince of Wales Hospital, Shatin, NT Hong Kong, China; 6 Shenzhen Research Institute, the Chinese University of Hong Kong, Shenzhen, Guangdong China; 7 Cancer Center, Union Hospital, Tongji Medical College, Huazhong University of Science and Technology, Wuhan, 430022 China; 8 Clinical Research Centre, Shenzhen People’s Hospital, the Second Clinical Medical College of Jinan University, Shenzhen, China; 9 Peking University Shenzhen Hospital, Shenzhen, Guangdong China; 10 Faculty of Medicine, Shenzhen University Health Science Center, Shenzhen University, Shenzhen, China

**Keywords:** NSCLC, FOXP3, EMT, Wnt, TCF4

## Abstract

**Background:**

The role of cancer cell FOXP3 in tumorigenesis is conflicting. We aimed to study FOXP3 expression and regulation, function and clinical implication in human non-small cell lung cancer (NSCLC).

**Methods:**

One hundred and six patients with histologically-confirmed NSCLC who underwent surgery were recruited for the study. Tumor samples and NSCLC cell lines were used to examine FOXP3 and its related molecules. Various cell functions related to tumorigenesis were performed. In vivo mouse tumor xenograft was used to confirm the in vitro results.

**Results:**

NSCLC patients with the high level of FOXP3 had a significant decrease in overall survival and recurrence-free survival. FOXP3 overexpression significantly induced cell proliferation, migration, and invasion, whereas its inhibition impaired its oncogenic function. In vivo studies confirmed that FOXP3 promoted tumor growth and metastasis. The ectopic expression of FOXP3 induced epithelial–mesenchymal transition (EMT) with downregulation of E-cadherin and upregulation of N-cadherin, vimentin, snail, slug, and MMP9. The oncogenic effects by FOXP3 could be attributed to FOX3-mediated activation of Wnt/β-catenin signaling, as FOXP3 increased luciferase activity of Topflash reporter and upregulated Wnt signaling target genes including c-Myc and Cyclin D1 in NSCLC cells. Co-immunoprecipitation results further indicated that FOXP3 could physically interacted with β-catenin and TCF4 to enhance the functions of β-catenin and TCF4, inducing transcription of Wnt target genes to promote cell proliferation, invasion and EMT induction.

**Conclusions:**

FOXP3 can act as a co-activator to facilitate the Wnt-b-catenin signaling pathway, inducing EMT and tumor growth and metastasis in NSCLC.

**Electronic supplementary material:**

The online version of this article (doi:10.1186/s12943-017-0700-1) contains supplementary material, which is available to authorized users.

## Background

FOXP3 has been studied in several types of cancer cells [1–7]. Most of these studies have shown the expression of FOXP3 is correlated with unfavourable prognosis, although there are some reports indicating the opposite role of FOXP3 [8, 9]. In in vitro and in vivo studies, FOXP3 has been widely reported as a suppressor gene in breast cancer [7, 10–12] and prostate cancer [3].

In lung cancer, Dimitrakopoulos et al. found that FOXP3 expression was correlated with lymph node metastasis [13]. Fu et al. reported that FOXP3 expression was associated with TNM stage and lymph node metastasis [14]. Tao et al. claimed FOXP3 alone had no prognostic value but a favourable prognostic value only when it was combined with T regulatory lymphocyte (Treg) counts [15]. Li et al. observed that FOXP3 was expressed in lung adenocarcinoma [16]. However, the function of FOXP3 in non-small cell lung cancer (NSCLC) has not been studied and the impact of FOXP3 on the Wnt/β-catenin pathway in cancers is unknown, Here, using both in vivo, in vitro models, we have demonstrated that FOXP3 is functional as an oncogenic molecule in NSCLC and have, for the first time, demonstrated that FOXP3 can act as a co-activator to facilitate the Wnt-catenin signaling pathway, inducing EMT and tumor growth and metastasis in NSCLC.

## Methods

### Cell lines and cultures

Human NSCLC cell lines, A549 and NCI-H460, were obtained from the American Type Culture Collection (ATCC, Manassas, VA), and cultured in RPMI-1640 medium. Human embryonic kidney epithelial cell line 293T cell line was purchased from Invitrogen, and cultured in DMEM medium. Lipofectamine 2000 (Invitrogen, Carlsbad, CA) was used to transfect cells with plasmid DNA. Total RNA and proteins were extracted from 80% confluent cells in culture dishes.

### NSCLC patient samples and immunohistochemistry

One hundred and six patients with histologically-confirmed NSCLC who underwent surgery at Prince of Wales Hospital between 2001 and 2007 with complete clinicopathologic characteristics (Additional file 1: Table S1) and follow-up data were enrolled in the study. Histologic type was determined according to the 2015 WHO classification [17]. Immunohistochemistry was performed on formalin-fixed paraffin sections according to standard protocols using two types of primary antibodies from Santa Cruz and Abcam, The tumor FOXP3 staining intensities were scored using the immunoreactive score (IRS) method [4] by a pathologist and an investigator. Tumor infiltrating FOXP3^+^ regulatory T-cells (Treg cells) were also counted at the same time. Human studies were approved by the Joint CUHK-NTEC Clinical Research Ethics committee.

### Western blot

The anti-E-Cadherin (CellSignaling, Danvers, MA, 1:2000), anti-N-Cadherin (Cellsignaling, 1:2000), anti-Vimentin (Abcam, 1:1000), anti-Snail (Cellsignaling, 1:2000), anti-Slug (Abcam, 1:2000), anti-MMP9 (Cellsignaling, 1:2000), anti-β-actin (Santa Cruz, 1:3000), anti-c-Myc (Santa Cruz, 1:1000), anti-Cyclin D1 (Santa Cruz, 1:1000), anti-TCF4 (Santa Cruz, 1:1000; Cellsignaling, 1:2000)), anti-LaminB1 (Cellsignaling, 1:2000), anti-FOXP3 (Abcam 1:3000, Cellsignaling 1:2000), anti-β-catenin (Cayman, Michigan, 1:5000), anti-GFP (Santa Cruz, 1:4000) were used as the primary antibodies. A 1:3000–5000 dilution of the HRP-linked anti-IgG (Santa Cruz) was used as the secondary antibody.

### Quantitative real-time PCR

Genes of interest were detected using SYBR® Premix Ex Taq ™ (Takara, Dalian, China) on Quantstudio™ 12k Flex Real-time PCR system (Applied Biosystems, Foster City, CA). The primer sequences are listed in Additional file 1: Table S2.

### Production of lentivirus for FOXP3 overexpression and knockdown

The full-length ORF (open reading sequence) of human FOXP3 gene (NM_014009.3) was PCR amplified from pcDNA3.1-FOXP3 and subcloned into the self-inactivating lentiviral vector PHIV-EGFP (Addgene) with an EF1-alpha promoter, an internal ribosome entry site (IRES) and EGFP. Lentiviral preparations were generated by transient transfection of HEK-293T cells by using pHIV-EGFP-FOXP3 (10 μg), pRSV-Rev. (2.2 μg), pMDLg/pRRE (4.72 μg), pMD2.G (3.08 μg).

FOXP3 shRNA was selected from the RNAi Consortium library (www.broadinstitute.org/rnai/public), which contains shRNAs against 15,000 human genes. We selected 5 highly scored target sequence against FOXP3 (NM_014009.3) mRNA sequence from the database: CCTCCACAACATGGACTACTT; CACACGCATGTTTGCCTTCTT; CTGAGTCTGCACAAGTGCTTT; TCCTACCCACTGCTGGCAAAT; TGTCCCTCACTCAACACAAAC. The corresponding oligoes generated by Invitrogen were subcloned into pLKO.1 (Addgene). Lentiviral preparations were generated as above except for that the transfection cocktail was replaced with pLKO.1 (6 μg), psPAX2 (4.5 μg) and pMD2.G (1.5 μg).

### Cell viability assay

Cell viability was determined by the MTT assay (ThermoFisher, Waltham, MA), and the absorbance was measured at 570 nm.

### Colony formation assay

One thousand cells were seeded and cultured for 10–14 days. Colonies (⩾50 cells/colony) were counted.

### Soft agar assay

Difco™ Agar (BD Biosciences, Erembodegem, Belgium) was used in the assay, 1000 cells per well were added in top agar and were cultured for 2 weeks. Colonies (⩾50 cells/colony) were counted.

### Wound healing assay

Scratch was made by a 200 μl pipette tip after cells were grown to 80% confluence. Cells were then incubated in medium containing 5% FBS. Gap size was measured 24 h later.

### Cell invasion assay

2.5 × 10^4^ cells were seeded in upper chamber with 300 μl medium containing 5% FBS. The lower chamber was filled with 1200 μl medium containing 10% FBS acting as chemotactic factors. After the incubation of 24–48 h, the migrated cells to the underside of the membrane were fixed and stained with 0.1% crystal violet.

### Immunofluorescence assay

The anti-E-Cadherin (Cellsignaling, 1:200) and anti-FOXP3 (Abcam, 1:100) were used as the primary antibodies. The cells were incubated with Alexa Fluor® 594 dye conjugated secondary antibody (ThermoFisher). The nucleus was stained by DAPI (ThermoFisher).

### Dual-luciferase reporter assay

HEK293T cells were plated in 24-well plates and co-transfected with various plasmids as indicated in the figures. Cells were collected 24 h after transfection, and luciferase activities were analyzed by the dual-luciferase reporter assay kit (Promega, Madison, WI). Reporter activity was normalized to the control Renilla.

### Gene expression microarrays and data analysis

Total RNA was extracted from A549-FOXP3 and A549-Control using TRIzol reagent (Invitrogen). The marked cRNAs were hybridized with the Agilent human whole genome gene expression Microarray (Agilent Technologies, Santa Clara, CA). Gene expression levels were standardized by the level of GAPDH. Differentially expressed genes were screened by the threshold of 2.0 fold-change and *p* value that was more than 0.05. Student’s t test was adopted for statistical analysis. Pathway analysis and Gene Ontology (GO) analysis were applied to determine the functions of those differentially expressed mRNAs by GO (www.geneontology.gov) [18] and the KEGG (Kyto Encyclopedia of Genes and Genomes) pathway database (http://www.genome.jp/kegg/pathway.html).

### Nuclear and cytoplasmic protein extraction

Cells were resuspended in 600 μl ice-cold Buffer I (1.5 mM MgCl2, 10 mM HEPES, 10 mM KCl, and protease inhibitor cocktail, pH 8.0), incubated on ice for 15 min and rotated once every 5 min. Then 10% Nonidet P-40 was added to a final 1% concentration. After a 10-s slight vortex, cells were centrifuged at 14,000 rpm for 3 min. Then the supernatants were collected as the cytoplasmic protein. The pellets were resuspended in 220 μl ice-cold Buffer II (420 mM NaCl, 20 mM HEPES, 0.2 mM EDTA, 1.5 mM MgCl2, 25% glycerol, and protease inhibitor cocktail, pH 8.0) and incubated on ice for 30 min. Then samples were centrifuged and the supernatants were transferred to new tubes as the nuclear fraction which was stored at −80 °C for later use.

### Co-immunoprecipitation assay

HEK-293T cells were co-transfected with the indicated plasmids with lipofectamine 2000 (Invitrogen), and the nuclear and cytoplasmic proteins were extracted as previously described [19, 20]. Three kinds of beads were used in this study for Co-IP assay: anti-FLAG M2 Magnetic Beads (Sigma-Aldrich, St Louis, MO); Pierce Anti-c-Myc Magnetic Beads (ThermoFisher); Protein A/G PLUS-Agarose (Santa Cruz). Briefly, the protein extracts were incubated with the equilibrated beads at 4 °C overnight with gentle mixing to capture the FLAG fusion proteins or Myc fusion proteins or specific antibody captured proteins. The magnetic beads or agarose beads were collected by placing the tube in the appropriate magnetic separator or by centrifuging. The beads were washed with TBS buffer to remove all of the non-specifically bounded proteins. The bounded fusion proteins were eluted from the beads with corresponding elution buffer for western blot analysis.

### In vivo tumor xenograft assays and metastasis assays

2 × 10^6^ A549-FOXP3 and A549-Control cells were separately subcutaneously inoculated into the left and right flank in the dorsal of the nude mice for in vivo xenograft assay. Tumor size was measured every 3 days for 18 days. The tumor volume (V) was calculated by the formula (length × width × width)/2. The tumors were excised and embedded in paraffin. For lung metastasis formation, 5 × 10^5^ A549-FOXP3 and A549-Control cells were injected into the lateral tail vein of the nude mice. Mice were euthanized 9 weeks after injection, and the lung, liver and spleen of each mice were subjected to formaldehyde fixation and followed by H&E staining. All experimental procedures were approved by the Animal Ethics Committee of the Chinese University of Hong Kong.

### Statistics

Continuous data were expressed as the median and range, discrete variables were presented as absolute values with relative frequencies. The independent Student’s t test was used to compare colony formation and gene expression between two groups. Paired t-test was used to compare the expression levels of FOXP3 in tumor tissues and adjacent normal tissues. Repeated Measures ANOVA was used to compare the tumor growth rate between two groups in the in vivo assay. The clinicopathologic features were compared using Pearson’s chi-squared test or Fisher’s exact test. Kaplan-Meier plots were used for OS and DFS rates, then compared with the log-rank test. Univariable or multivariable Cox proportional hazard regression was performed to evaluate the predictive values of FOXP3 and other clinicopathologic features. A two-tailed *p* value less than 0.05 was considered statistical significance. All the tests were performed by SPSS version 16.0.

## Results

### FOXP3 is highly expressed in NSCLC and correlated with poor prognosis

**Fig. 1:**
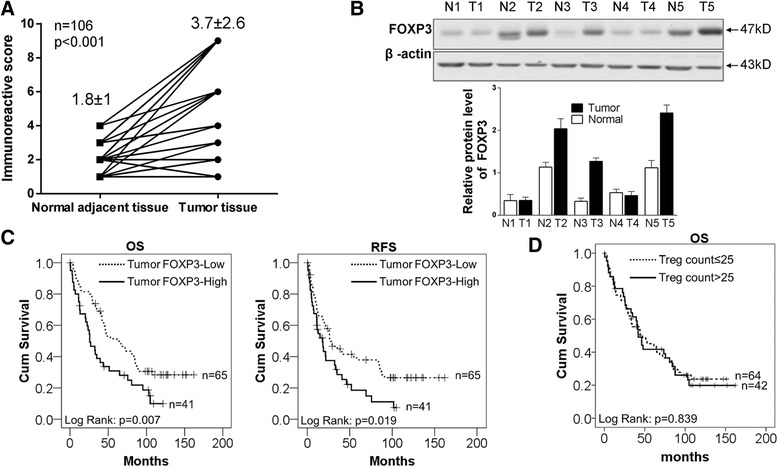
FOXP3 is highly expressed in NSCLC and correlated with poor prognosis. **a** The levels of FOXP3 in 106 paired NSCLC tissues and adjacent normal tissues were scored by IRS method (5) and analyzed by paired t-test. Mean ± SD is shown in the figure. For images of the tissue staining, please refer to Additional file 2: Figure S1. **b** FOXP3 protein levels were higher in tumor than in peritumoral tissues, as determined by western blot. **c** Kaplan–Meier representation of the overall survival and recurrence-free survival of the two groups of patients with high (*n* = 41, solid line) or low (*n* = 65, dotted line) FOXP3 expression in NSCLC tissues. Statistical analysis was performed with the log-rank test. **d** Kaplan–Meier representation of the overall survival of the two groups of patients with Treg cell counts >25 (*n* = 42, solid line) or ≤25 (*n* = 64, dotted line) in NSCLC tissues. Statistical analysis was performed with the log-rank test

To determine whether FOXP3 was expressed in NSCLC and correlated with patient prognosis, we performed immunohistochemistry on NSCLC tissues using two types of monoclonal anti-FOXP3 antibodies. FOXP3 immunostaining was seen in NSCLC cells and Treg cells (Additional file 2: Figure S1). Tumor FOXP3 expression mainly exhibited a mixture of diffuse staining in the cytoplasm, the nucleus or in both of them. The FOXP3 expression levels in NSCLC were scored 1 to 12 according to the IRS method. Specimens scored 1–4 were classified as ‘Tumor FOXP3-Low’ and 6–12 as ‘Tumor FOXP3-High’. In 106 NSCLC specimens, 41 cancer tissues were detected high FOXP3 expression and 65 cancer tissues showed low FOXP3 expression. Statitically, the overall expression of FOXP3 was much higher in cancer tissues than in normal adjacent tissues (*p* < 0.001) (Fig. 1a). To confirm this finding, western blotting was performed to detect their protein levels in 5 randomly selected paired NSCLC specimens. As shown in Fig. 1b, 3 out of 5 showed higher level of FOXP3 protein in tumor tissues than that in adjacent normal tissues.

We analyzed the correlation between clinicopathologic characteristics and tumor FOXP3 expression, the results showed that there was no significant association of FOXP3 expression levels with clinicopathologic characteristics. However, we found a correlation between tumor FOXP3 expression and Treg cell counts (Additional file 1: Table S3).

The correlation of FOXP3 expression with the long-term survival rate after surgery operation was analyzed. Kaplan–Meier analysis showed that patients with high levels of FOXP3 expression in tumor had shorter overall survival time than those with low levels of FOXP3 expression (*P* < 0.05, Fig. 1c). The mean overall survival time of patients with high FOXP3 expression in tumor tissues was 44.3 ± 6.54 months, while that with low FOXP3 expression level was 78.1 ± 7.35 months (*P* = 0.007).

Kaplan–Meier analysis also showed that patients with high levels of FOXP3 expression in tumor had shorter recurrence-free survival time than those with low levels of FOXP3 expression (*P* < 0.05, Fig. 1c). The mean recurrence-free survival time of the patients with high FOXP3 expression in tumor tissues was 30.5 ± 5.47 months, while those with low FOXP3 expression was 63.8 ± 8.16 months (*P* = 0.019).

To further explore whther FOXP3 had any prognostic value, we conducted Cox proportional Hazard regression analysis of patients’ overall survival and recurrence-free survival. In multivariate analysis of overall survival, when other risk factors such as histology grade, pathology stage, pathology tuberculosis and nodal status were taken in consideration, the HR of high FOXP3 expression was improved from 1.86 (95% CI: 1.18–1.92, *P* = 0.01) to 2.09 (95% CI: 1.27–3.44, *P* = 0.00) (Additional file 1: Table S4). In multivariate analysis of recurrence-free survival, when other risk factors were taken in consideration, the HR of high FOXP3 expression was improved from 1.71, (95% CI: 1.09–2.70, *P* = 0.02) to 1.83 (95% CI: 1.11–3.02, *P* = 0.02) (Additional file 1: Table S5). Both univariate analyses and multivariate analyses indicate that FOXP3 is an independent predictor for overall survival and recurrence-free survival of NSCLC patients.We also analyzed the Treg cells in NSCLC. Patients were divided into two groups according Treg cell numbers, we found that Treg cells alone did not have a prognostic value in NSCLC (*P* > 0.05, Fig. 1d).

### Ectopic expression of FOXP3 promotes tumourigenic properties of NSCLC cells

**Fig. 2:**
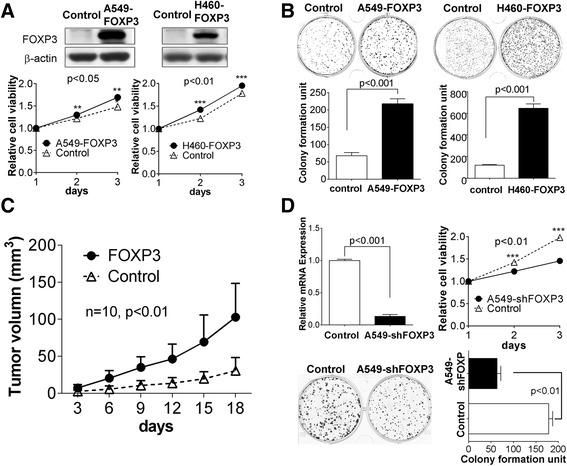
FOXP3 promotes tumor growth in NSCLC. **a** Effect of FOXP3 on cell viability was evaluated by MTT assay in A549 and H460 cells. **b** Effect of FOXP3 on colony formation in A549 and H460 cells. **c** Subcutaneous tumor growth curve of A549-FOXP3 cells in nude mice was compared with control cells. The FOXP3 group showed an enhanced tumor growth compared with the control group (*P* < 0.05). **d** Effects of FOXP3 knockdown on cell viability and colony formation in A549 cells

We examined the effect of ectopic expression of FOXP3 on the several tumorigentic features of cancer cells using NSCLC cell lines (A549 and H460). FOXP3 lentivirus was used to improve transfection efficiency, and the expression of FOXP3 was validated by western blot (Fig. 2a). Ectopic expression of FOXP3 caused a significant increase in cell viability in both A549 (*p* < 0.001) and H460 (*p* < 0.001) cells (Fig. 2a). This promoting effect was further confirmed by the colony formation assay, in which FOXP3 significantly increased the colony formation in A549 (*p* < 0.001) and H460 (*p* < 0.001) when compared with control (Fig. 2b).

Since FOXP3 promotes cell growth in vitro, we next tested whether FOXP3 could promote the growth of NSCLC cells in nude mice. 18 days after inoculation we found that the mean volume of tumors infected with FOXP3 was 2.6-fold larger than that of infected with control virus, which formed either no tumor or much smaller tumors. The tumor growth curves of A549-FOXP3 and A549-Control in nude mice were shown in Fig. 2c and Additional file 2: Figure S2. In contrast to A549-control, A549-FOXP3 exhibited markedly increased growth rate in the xenograft model (*p* < 0.05). Taken together, these findings have demonstrated that FOXP3 can exhibit obvious tumorigenic functions in NSCLC.

To further validate the oncogenic effect of FOXP3 on the growth of NSCLC, shFOXP3 lentivirus was generated and the silence effect was validated by qPCR (Fig. 2d). MTT assay showed that the knockdown of FOXP3 in A549 could inhibit cell growth (*p* < 0.05) (Fig. 2d). The colony formed by shFOXP3 lentivirus-infected cells was fewer than the control lentivirus-infected cells (Fig. 2d). These results further support that FOXP3 is an oncogenic molecule in NSCLC.

### Ectopic expression of FOXP3 induces EMT in NSCLC cells

**Fig. 3:**
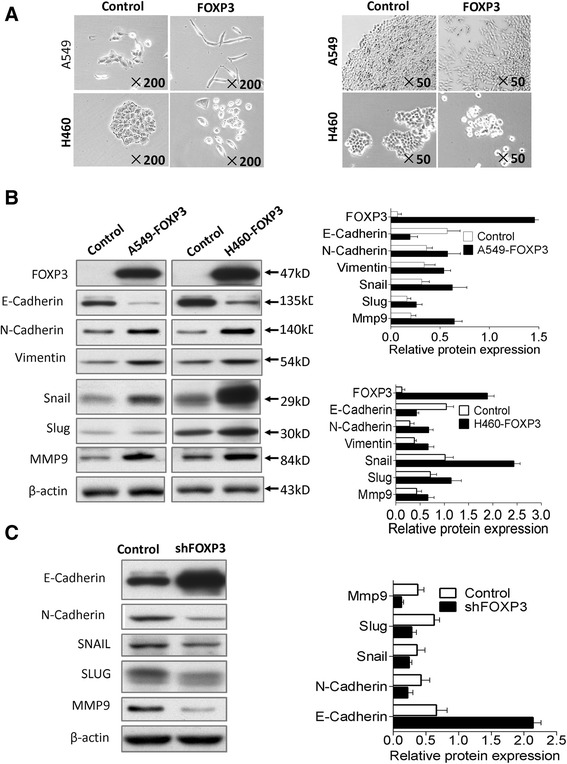
FOXP3 induces EMT in NSCLC cells. **a** FOXP3 induces mesenchymal morphology changes in A549 and H460 cells: spindle shape and loss of cell-cell contact. **b** The effect of FOXP3 overexpression on EMT marker expression was assessed by western blot. **c** Effect of FOXP3 knockdown on EMT marker expression in A549 cells

When we first infected A549 cells by FOXP3 lentivirus, we found a significant spindle shape change in A549 cells (Fig. 3a) compared to control cells, which is further confirmed in H460 cells infected with FOXP3 lentivirus (Fig. 3a). When we seeded A549-FOXP3 cells in 6-well plate to form colony for about 1–2 weeks, it was found that cells lost cell-cell contact and formed a scattered phenomenon (Fig. 3a). A similar finding was also observed in H460-FOXP3 cells after two-week growth (Fig. 3a). Spindle shape change and lose of cell-cell contact are the characteristics of cell morphology change during Epithelial-Mesenchymal Transition [21]. E-Cadherin is the most remarkable markers of EMT, immunofluorescent staining demonstrated that E-Cadherin was markedly reduced in A549-FOXP3 cells compared with control cells (Additional file 2: Figure S3), which is consistent with the cell morphology change that FOXP3 induced the EMT. Western blot was used to analyze EMT markers induced by FOXP3 in A549 and H460 cells (Fig. 3b). E-Cadherin was downregulated in both A549-FOXP3 and H460-FOXP3 cells compared to control cells, which is consistent with our immunofluorescent staining assays. An upregulation of N-Cadherin, vimentin, Snail, Slug and MMP9 was also induced by FOXP3 in both cells. All these changes in biomarkers suggest a transition of epithelial cells to mesenchymal cells.

Then, we knocked down FOXP3 to check whether it could reverse the change of EMT markers induced. A549-shFOXP3 cells which was generated early was used for analysis. Western blot results (Fig. 3c) showed that E-Cadherin was upregulated, N-Cadherin, Vimentin, snail, slug and MMP9 were downregulated compared to control cells. These changes were opposite to FOXP3 overexpression experiment, and further validated the role FOXP3 on EMT induction.

### Ectopic expression of FOXP3 promotes cancer metastasis in vitro and in vivo

**Fig. 4:**
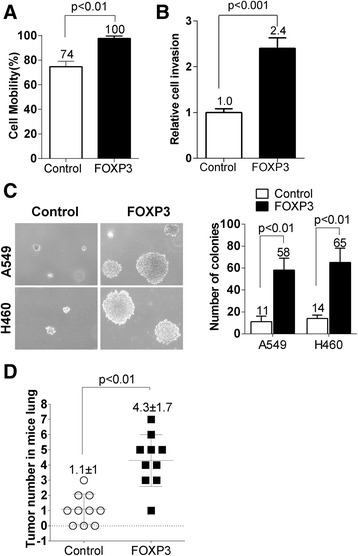
FOXP3 promotes tumor metastasis in NSCLC. **a** Effect of FOXP3 on cell migration was evaluated by wound healing assay in A549 cells. FOXP3-expressing A549 cells had a much stronger healing ability than control cells (*p* < 0.01). **b** Effect of FOXP3 on cell invasion was evaluated in A549 cells by Matrigel invasion assay. FOXP3-expressing A549 cells showed higher penetration rate through the Matrigel-coated membrane compared with control cells (*p* < 0.001). **c** Soft agar growth analysis demonstrated that FOXP3-expressing A549 and H460 cells had a stronger tumorigenicity than control cells (*p* < 0.01). **d** FOXP3 promotes tumor metastasis in vivo. The total number of metastatic nodules was quantified in lungs of nude mice 8 weeks after tail vein injection of control and FOXP3-expressing A549 cells. Values for individual mouse were dotted in the plot and values by group were also denoted. Data are mean ± s.d. Please refer to Additional file 2: Figure S4 for representative images for **a**, **b** and **d**

First, the result of the wound healing assay showed that cells infected with FOXP3 lentivirus had a significant healing ability than control cells in wound formation, which is about 1.35 fold faster than control cells (*p* < 0.01) (Fig. 4a, Additional file 2: Figure S4A), suggesting A549-FOXP3 has an stronger ability in migration than A549-control. Second, we did Matrigel invasion assays to test the ability of invasion. Photomicrographs and histograms (Fig. 4b) indicated that the number of A549-FOXP3 cells migrated to lower chambers through Matrigel is more than that of control cells migrated to the lower chambers (2.4 fold, *p* < 0.01). Third, soft agar assays showed that A549-FOX3 and H460-FOXP3 cells formed more and larger colonies (*p* < 0.01) compared to control cells (Fig. 4c). Finally, to test whether FOXP3 could promote metastasis in vivo, A549-FOXP3 and control cells were inoculated into nude mice via tail vein injection. The mice were sacrificed 2 months later to examine metastatic nodules in lung, liver and spleen. The results of H&E staining showed that ectopic expression of FOXP3 in A549 cells led to a significant increase in the total number of metastatic nodules in the lung of nude mice (Fig. 4b-d), although we didn’t find metastatic nodules in the liver and spleen of nude mice (data not shown). All these findings suggest that FOXP3 is able to promote metastasis in NSCLC cells.

### FOXP3 enhances Wnt/β-catenin pathway in NSCLC

To gain insights into the mechanism of FOXP3-mediated tumorigenicity and EMT, microarray gene expression profiling was conducted. Hierarchical clustering analysis showed that FOXP3 overexpression led to a clear and consistent difference in the gene expression profile (Additional file 2: Figure S5). We identified 1811 upregulated genes, and 2292 downregulated genes (data not shown). We conducted GO analysis to reveal general functional features implemented by FOXP3 in A549 cells using upregulated genes. The results of both Biological process analysis (Additional file 2: Figure S6A) and cellular component analysis (Additional file 2: Figure S6B) coincided with the characteristics of EMT process.

**Fig. 5:**
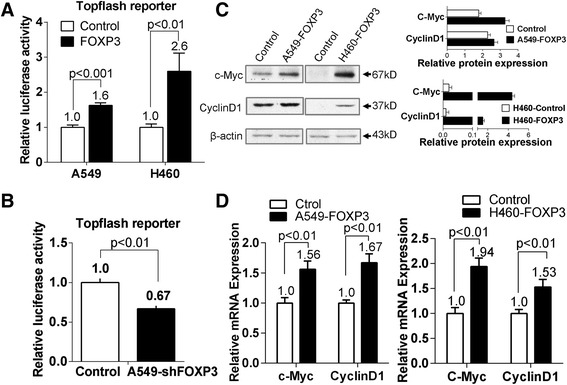
The oncogenic effect of FOXP3 is mediated by activating Wnt/β-catenin signaling pathway. **a** The effect of FOXP3 on Wnt/β-catenin signaling pathway was assessed by dual-luciferase reporter assays in A549 and H460 cells. **b** Knockdown of FOXP3 impaired Topflash reporter activities in A549 cells. **c** FOXP3 upregulated protein expression of c-Myc and Cyclin D1 in A549 and H460 cells. **d** FOXP3 upregulated mRNA expression of c-Myc and Cyclin D1 in A549 and H460 cells

Pathway analysis suggests multiple pathways might be involved in A549 cells after FOXP3 stimulation (Additional file 2: Figure S7). Wnt/β–catenin pathway was selected for further studies as it is broadly involved in the process of promoting tumorigenicity, cell stemness and EMT induction [21, 22]. Data analysis of Wnt/β–catenin pathway (data not shown) suggested that 4 out of 6 direct downstream genes including c-Myc, Cyclin D1, c-Jun, fra-1 were upregulated, the finding of which is another evidence to support an activated Wnt/β–catenin pathway in A549-FOXP3 cells. To determine whether Wnt/β–catenin pathway was activated by FOXP3 in NSCLC cells, we first performed a luciferase experiment with Top-Luc Flash reporter and pRL-TK (as an internal control). Our results showed that FOXP3 increased luciferase activity dramatically in both A549 and H460 cells (Fig. 5a). And we observed a significant reduction of the luciferase activity in A549 cells when FOXP3 was silenced by lentivirus (Fig. 5b). These results suggest that the overexpression of FOXP3 stimulates the Wnt/β–catenin pathway in NSCLC cells.

We also analyzed the expression of c-Myc and Cylin D1, two well-known target genes of the Wnt/β–catenin pathway [23–25], in A549-FOXP3 and H460-FOXP3 cells. Both qPCR (Fig. 5d) and western blot (Fig. 5c) results showed the elevated expression of c-Myc and Cylin D1.

### FOXP3 acts as a co-activator of Wnt/β-catenin signaling in NSCLC cells

**Fig. 6:**
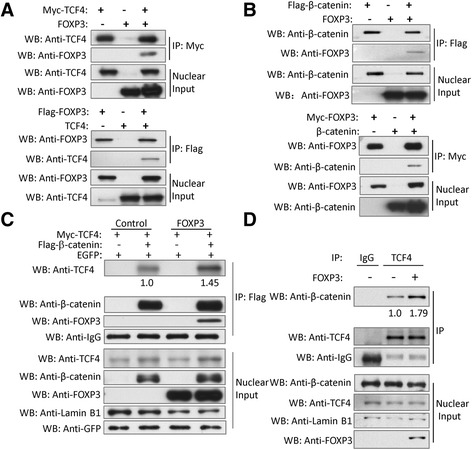
FOXP3 acts as a co-activator of Wnt/β-catenin signaling in NSCLC cells. **a** Co-immunoprecipitation showed the physical interaction between FOXP3 and TCF4 in HEK-293 cells co-expressing Myc-TCF4 and FOXP3 or Flag-FOXP3 and TCF4. **b** Co-immunoprecipitation showed the physical interaction between FOXP3 and β-catenin in HEK-293 cells co-expressing Flag-β-catenin and FOXP3 or Myc-FOXP3 and β-catenin. **c** FOXP3 enhances the interaction of β-catenin and TCF4. Plasmids of Myc-TCF4, Flag-β-catenin and FOXP3 were co-transfected into HEK293T cells according to the situations indicated above. EGFP plasmids was co-transfected as the control of transfection efficiency. The cell lysates were subjected to IP with an anti-Flag antibody. The immunoprecipitates and the nuclear proteins were subjected to Western blots using the indicated antibodies. Lamin B1 was used to be the loading control of the nuclear protein. **d** FOXP3 enhances the interaction of endogenous TCF4 and β-catenin. A549 cells were infected with FOXP3 lentivirus or control lentivirus as indicated above for 48 h. The nuclear fractions were incubated with an anti-TCF4 antibody for the IP experiment. IgG was used as a negative control. The immunoprecipitates and the nuclear extracts were subjected to Western blots using the indicated antibodies. Lamin B1 was used to be the loading control of the nuclear protein

In most cases, the elevated expression of β-catenin is the cause of activating canonical pathway. However, our western blot results did not show the significant changes in β-catenin levels in A549-FOXP3 and H460-FOXP3 cells compared with corresponding control cells. Since our immunofluorescent staining results (Additional file 2: Figure S8) which showed the localization of overexpressed FOXP3 protein was mainly in the nucleus of A549-FOXP3 and H460-FOXP3 cells, it is likely that the oncogenic function of FOXP3 is correlated with the LEF/TCF complex, which is the potent Wnt/β-catenin transcriptional factor in the nucleus [23, 26]. We thus examined whether FOXP3 interacted with the nuclear components of the Wnt/β-catenin signaling pathway. Reciprocal immunoprecipitation experiments indicated that FOXP3 and TCF4 interacted with each other in HEK-293T cells (Fig. 6a), suggesting that FOXP3 could form a complex with TCF4 in the nucleus. In the canonical pathway, β-catenin combined with LEF/TCF4 complex to activate Wnt signaling [19, 20, 27, 28]. We thus questioned whether FOXP3 could also form a complex with β-catenin. The Co-IP test demonstrated that β-catenin and FOXP3 could precipitate down each other in the nucleus (Fig. 6b), suggesting that FOXP3 could also associate with β-catenin to form a complex. To this end, we speculated FOXP3 might enhance the formation of β-catenin·TCF4 complex and work as a co-activator of Wnt/β-catenin pathway. Immunoprecipitation results showed that the interaction of FLAG-β-catenin with Myc-TCF4 increased 1.45 fold in the nucleus when FOXP3 was co-expressed (Fig. 6c). Furthermore, an immunoprecipitation experiment using endogenous proteins demonstrated that the interaction of endogenous β-catenin and endogenous TCF4 was significantly increased 1.79 fold in the nucleus by FOXP3 overexpression (Fig. 6d). Taken together, these results suggest that FOXP3 is a co-activator to enhance the formation of the β-catenin and TCF4 complex in the nucleus of NSCLC cells.

## Discussion

In this study, we found that tumor FOXP3 expression was frequently upregulated in NSCLC tissues, and in most cases showed a mixture of cytoplasmic and nuclear expression. However, tumor FOXP3 expression was not statistically correlated with any clinicopathologic features, though advanced pathologic stage (III-IV) seemed to be associated with high FOXP3 expression. Tumor FOXP3 has been claimed as an independent prognostic indicator for tongue squamous cell carcinoma [2] and breast cancer [29]. However, to our best of knowledge it has not been reported as an independent prognostic factor in NSCLC. In this study, we found that high tumor FOXP3 expression was significantly correlated with worse overall survival and recurrence-free survival of the patients, and showed independent prognostic value in NSCLC. FOXP3 expression in Treg cells could also be seen in NSCLC specimens. Although Treg cells in NSCLC were reported with poor prognosis in some publications [30, 31]. However, we did not find its prognostic value in NSCLC. Tumor FOXP3 expression has a positive correlation with Treg cell counts in our NSCLC specimens. Thus we speculate that tumor FOXP3 may be a confounding factor to affect the prognostic value in NSCLC.

In vitro and in vivo assays demonstrated the oncogenic role of FOXP3 in NSCLC. Ectopic expression of FOXP3 contributed to tumor growth and metastasis in NSCLC cells (A549 and H460) by promoting cell proliferation, migration and invasion as evidenced by cell viability assay, colony formation assay, soft agar assay, wound healing assay and transwell assay. The promotion of tumor growth and metastasis by FOXP3 was further confirmed in subcutaneous xenograft tumor mouse model and tail-vein-injection metastatic mouse model. On the other hand, lentivirus-mediated knockdown of FOXP3 significantly inhibited cell proliferation and clonogenicity. Moreover, we observed that ectopic expression of FOXP3 in A549 and H460 led to downregulation of E-Cadherin and upregulation of N-Cadherin, vimentin, Snail, Slug and MMP9, as well as the mesenchymal specific morphology changes: spindle shape, loss of cell-cell contact and cell scattering. All these changes in biomarkers and morphology suggest a transition of epithelial cells to mesenchymal cells. This transition was further confirmed by microarray analysis of biological process and cellular component. On the other hand, the knockdown of FOXP3 impairs its ability in downregulating E-Cadherin and upregulating N-Cadherin, Snail, Slug and MMP9. This is the first report on the role of FOXP3 in EMT induction.

FOXP3 has been reported as a suppressor gene in breast cancer [7, 10–12] and prostate cancer [3] via repressing the expression of oncogene such as HER2, SKP2, p21, LATS2 and c-Myc. Using qPCR method, we did not see the similar changes of these molecules in NSCLC cells when FOXP3 was overexpressed (data not shown). Conversely, the pathway analysis of the gene expression profiling indicated an activated HER2 signaling pathway in FOXP3-overexpression NSCLC cells. The findings of Western blot and qPCR verified the upregulation of c-Myc in FOXP3-overexpression NSCLC cells. EMT is an important step in the progression of cancer metastasis and invasion [32], and also contribute to the increase in resistance to pro-apoptotic and chemotherapeutic drugs and the induction of cancer cell stemness [33]. Our results have suggested that FOXP3 can function as an oncogene in NSCLC that has a different genetic background from breast cancer and prostate cancers. Our finding is supported by the studies reported by others, demonstrating that the overexpression of FOXP3 decreases mouse Lewis lung cancer cell sensitivity to chemotherapy and that the ectopic expression of FOXP3 promotes cell growth, migration and invasion in lung adenocarcinoma [16]. In some other types of cancers [34, 35], FOXP3 has also been shown to promote cancer growth, migration and invasion.

We further elucidated the downstream signaling pathway responsible for FOXP3 oncogenic function in NSCLC and found that the FOXP3-mediated tumor growth and metastasis could be, at least partly, attributed to the activation of Wnt/β-catenin signaling, which is critical for the initiation and progression of NSCLC [36, 37]. The FOXP3-induced Wnt/β-catenin signaling in A549 and H460 was evidenced by the high luciferase activity of Topflash reporter, and the upregulation of Wnt signaling target gene (c-Myc and Cyclin D1) expression. Moreover, the knockdown of FOXP3 impaired the Topflash reporter luciferase activity. The activation of Wnt/β-catenin signaling pathway can also contribute to the induction of EMT via stimulating several EMT-related transcription factors, such as Snail, Slug, Twist, ZEB1, ZEB2 and E47 [38].

FOXP3 has constantly been regarded as a transcriptional factor functioned in cancer cells [11, 39–41]. However its role in the regulation of Wnt/β-catenin signaling pathway in human cancers is unknown. Our Co-IP result indicated that FOXP3 could interact with β-catenin and TCF4 respectively and reciprocally, and enhance the function of β-catenin and TCF4 in the nucleus.

## Conclusion

FOXP3 may act to facilitate the formation of β-catenin-TCF4 complex and enhance their function to activate EMT-related molecules such as snail and slug, leading to the induction of EMT and the promotion of NSCLC growth and metastasis (Additional file 2: Figure S9).

## Additional files

Additional file 1: Table S1. Clinical characteristics of NSCLC patients. **Table S2.** DNA sequences of real-time PCR primers used for the detection of mRNA expression. **Table S3.** Clinical characteristics and distribution of patients according to their FOXP3 expression levels. **Table S4.** Cox proportional Hazard regression analysis of patients’ overall survival. **Table S5.** Cox proportional Hazard regression analysis of patients’ recurrence-free survival. (PDF 392 kb)

Additional file 2: Supplementary Methods and Figures. (DOCX 3930 kb)

## Abbreviations

EMT: Epithelial–mesenchymal transition
GO: Gene Ontology
IRES: Internal ribosome entry site
IRS: Immunoreactive score
NSCLC: Small cell lung cancer
Treg: T regulatory lymphocyte
Treg cells: Tumor infiltrating FOXP3+ regulatory T-cells
V: Tumor volume

## References

[1] Hinz S, Pagerols-Raluy L, Oberg HH, Ammerpohl O, Grussel S, Sipos B, Grützmann R, Pilarsky C, Ungefroren H, Saeger HD (2007). Foxp3 expression in pancreatic carcinoma cells as a novel mechanism of immune evasion in cancer. Cancer Res.

[2] Liang YJ, Liu HC, Su YX, Zhang TH, Chu M, Liang LZ, Liao GQ (2011). Foxp3 expressed by tongue squamous cell carcinoma cells correlates with clinicopathologic features and overall survival in tongue squamous cell carcinoma patients. Oral Oncol.

[3] Wang L, Liu R, Li W, Chen C, Katoh H, Chen GY, McNally B, Lin L, Zhou P, Zuo T (2009). Somatic single hits inactivate the X-linked tumor suppressor FOXP3 in the prostate. Cancer Cell.

[4] Weller P, Bankfalvi A, Gu X, Dominas N, Lehnerdt GF, Zeidler R, Lang S, Brandau S, Dumitru CA (2014). The role of tumour FoxP3 as prognostic marker in different subtypes of head and neck cancer. Eur J Cancer.

[5] Winerdal ME, Marits P, Winerdal M, Hasan M, Rosenblatt R, Tolf A, Selling K, Sherif A, Winqvist O (2011). FOXP3 and survival in urinary bladder cancer. BJU Int.

[6] Zhang H, Sun H (2010). Up-regulation of Foxp3 inhibits cell proliferation, migration and invasion in epithelial ovarian cancer. Cancer Lett.

[7] Zuo T, Liu R, Zhang H, Chang X, Liu Y, Wang L, Zheng P, Liu Y (2007). FOXP3 is a novel transcriptional repressor for the breast cancer oncogene SKP2. J Clin Investig.

[8] Ma GF, Miao Q, Liu YM, Gao H, Lian JJ, Wang YN, Zeng XQ, Luo TC, Ma LL, Shen ZB (2014). High FoxP3 expression in tumour cells predicts better survival in gastric cancer and its role in tumour microenvironment. Br J Cancer.

[9] Suh JH, Won KY, Kim GY, Bae GE, Lim SJ, Sung JY, Park YK, Kim YW, Lee J (2015). Expression of tumoral FOXP3 in gastric adenocarcinoma is associated with favorable clinicopathological variables and related with hippo pathway. Int J Clin Exp Pathol.

[10] Li W, Wang L, Katoh H, Liu R, Zheng P, Liu Y (2011). Identification of a tumor suppressor relay between the FOXP3 and the hippo pathways in breast and prostate cancers. Cancer Res.

[11] Liu R, Wang L, Chen G, Katoh H, Chen C, Liu Y, Zheng P (2009). FOXP3 up-regulates p21 expression by site-specific inhibition of Histone Deacetylase 2/Histone Deacetylase 4 association to the locus. Cancer Res.

[12] Zuo T, Wang L, Morrison C, Chang X, Zhang H, Li W, Liu Y, Wang Y, Liu X, Chan MW (2007). FOXP3 is an X-linked breast cancer suppressor gene and an important repressor of the HER-2/ErbB2 Oncogene. Cell.

[13] Dimitrakopoulos F, Papadaki H, Antonacopoulou AG, Kottorou A, Gotsis AD, Scopa C, Kalofonos HP, Mouzaki A (2011). Association of FOXP3 expression with non-small cell lung cancer. Anticancer Res.

[14] Fu HY, Li C, Yang W, Gai XD, Jia T, Lei YM, Li Y (2013). FOXP3 and TLR4 protein expression are correlated in non-small cell lung cancer: implications for tumor progression and escape. Acta Histochem.

[15] Tao H, Mimura Y, Aoe K, Kobayashi S, Yamamoto H, Matsuda E, Okabe K, Matsumoto T, Sugi K, Ueoka H (2012). Prognostic potential of FOXP3 expression in non-small cell lung cancer cells combined with tumor-infiltrating regulatory T cells. Lung Cancer.

[16] Li Y, Li D, Yang W, Fu H, Liu Y, Li Y (2015). Overexpression of the transcription factor FOXP3 in lung adenocarcinoma sustains malignant character by promoting G1/S transition gene CCND1. Tumour Biol.

[17] Travis WD, Brambilla E, Nicholson AG, Yatabe Y, Austin JHM, Beasley MB, Chirieac LR, Dacic S, Duhig E, Flieder DB (2015). The 2015 World Health Organization classification of lung tumors. J Thorac Oncol.

[18] Richards JS (2005). Ovulation: new factors that prepare the oocyte for fertilization. Mol Cell Endocrinol.

[19] Turley EA, Veiseh M, Radisky DC, Bissell MJ (2008). Mechanisms of disease: epithelial–mesenchymal transition—does cellular plasticity fuel neoplastic progression?. Nat Clin Pract Oncol.

[20] Craene BD, Berx G (2013). Regulatory networks defining EMT during cancer initiation and progression. Nat Rev Cancer.

[21] Mosimann C, Hausmann G, Basler K (2009). β-catenin hits chromatin: regulation of Wnt target gene activation. Nat Rev Mol Cell Biol.

[22] Teo J, Kahn M (2010). The Wnt signaling pathway in cellular proliferation and differentiation: a tale of two coactivators. Adv Drug Deliv Rev.

[23] Veeman MT, Axelrod JD, Moon RT (2003). A second canon: functions and mechanisms of beta-catenin-independent Wnt signaling. Dev Cell.

[24] Cadigan KM, Waterman ML (2012). TCF/LEFs and Wnt signaling in the nucleus. Cold Spring Harb Perspect Biol.

[25] Duchartre Y, Kim Y, Kahn M (2016). The Wnt signaling pathway in cancer. Crit Rev Oncol Hematol.

[26] van Loosdregt J, Fleskens V, Tiemessen MM, Mokry M, van Boxtel R, Meerding J, Pals CE, Kurek D, Baert MR, Delemarre EM (2013). Canonical Wnt signaling negatively modulates regulatory T cell function. Immunity.

[27] Wu Y, Zhang Y, Zhang H, Yang X, Wang Y, Ren F, Liu H, Zhai Y, Jia B, Yu J (2010). p15RS attenuates Wnt/{beta}-catenin signaling by disrupting {beta}-catenin TCF4 interaction. J Biol Chem.

[28] Zhang Y, Liu C, Duan X, Ren F, Li S, Jin Z, Chang Z (2014). CREPT/RPRD1B, a recently identified novel protein highly expressed in tumors, enhances the -catenin{middle dot}TCF4 transcriptional activity in response to Wnt signaling. J Biol Chem.

[29] Merlo A, Casalini P, Carcangiu ML, Malventano C, Triulzi T, Mènard S, Tagliabue E, Balsari A (2009). FOXP3 expression and overall survival in breast cancer. J Clin Oncol.

[30] O’Callaghan DS, Rexhepaj E, Gately K, Coate L, Delaney D, O’Donnell DM, Kay E, O’Connell F, Gallagher WM, O’Byrne KJ (2015). Tumour islet Foxp3+ T-cell infiltration predicts poor outcome in nonsmall cell lung cancer. Eur Respir J.

[31] Tao H, Shien K, Soh J, Matsuda E, Toyooka S, Okabe K, Miyoshi S (2014). Density of tumor-infiltrating FOXP3+ T cells as a response marker for induction Chemoradiotherapy and a potential prognostic factor in patients treated with Trimodality therapy for locally advanced non-small cell lung cancer. Ann Thorac Cardiovasc Surg.

[32] Savagner P (2001). Leaving the neighborhood: molecular mechanisms involved during epithelial-mesenchymal transition. BioEssays.

[33] Singh A, Settleman J (2010). EMT, cancer stem cells and drug resistance: an emerging axis of evil in the war on cancer. Oncogene.

[34] Chu R, Liu SYW, Vlantis AC, van Hasselt CA, Ng EKW, Fan MD, Ng SK, Chan AB, Du J, Wei W (2015). Inhibition of Foxp3 in cancer cells induces apoptosis of thyroid cancer cells. Mol Cell Endocrinol.

[35] Luo Q, Zhang S, Wei H, Pang X, Zhang H (2015). Roles of Foxp3 in the occurrence and development of cervical cancer. Int J Clin Exp Pathol.

[36] Segditsas S, Tomlinson I (2006). Colorectal cancer and genetic alterations in the Wnt pathway. Oncogene.

[37] Yang J, Chen J, He J, Li J, Shi J (2016). Wnt signaling as potential therapeutic target in lung cancer. Expert Opin Ther Targets.

[38] Puisieux A, Brabletz T, Caramel J (2014). Oncogenic roles of EMT-inducing transcription factors. Nat Cell Biol.

[39] Li WQ, Katoh H, Wang LZ, Yu XC, Du ZW, Yan XL, Zheng P, Liu Y (2013). FOXP3 regulates sensitivity of cancer cells to irradiation by transcriptional repression of BRCA1. Cancer Res.

[40] Liu R, Liu C, Chen D, Yang WH, Liu X, Liu CG, Dugas CM, Tang F, Zheng P, Liu Y (2015). FOXP3 controls an miR-146/NF-кB negative feedback loop that inhibits apoptosis in breast cancer cells. Cancer Res.

[41] Liu R, Yi B, Wei S, Yang WH, Hart KM, Chauhan P, Zhang W, Mao X, Liu X, Liu CG (2015). FOXP3-miR-146-NF-кB Axis and therapy for precancerous lesions in prostate. Cancer Res.

