# Supplementary material for: FOXP3 promotes tumor growth and metastasis by activating Wnt/β-catenin signaling pathway and EMT in non-small cell lung cancer
Source: Mol Cancer. 2017 Jul 17;16:124. doi: 10.1186/s12943-017-0700-1 (PMC5514503; doi:10.1186/s12943-017-0700-1)
Supplement: Supplementary file 1 — Clinical characteristics of NSCLC patients. Table S2. DNA sequences of real-time PCR primers used for the detection of mRNA expression. Table S3. Clinical characteristics and distribution of patients according to their FOXP3 expression levels. Table S4. Cox proportional Hazard regression analysis of patients’ overall survival. Table S5. Cox proportional Hazard regression analysis of patients’ recurrence-free survival. (PDF 392 kb) [file 12943_2017_700_MOESM1_ESM.pdf]

Table S1. Clinical characteristics of NSCLC patients

Characteristics		number	%
All		106	
Age		66.7±11.6	
Gender			
	Male	75	71
	Female	31	29
Smoking status			
	smoker	55	52
	non-smoker	51	48
Histology			
	squamous cell carcinoma	27	25
	adenocarcinoma	64	60
	large cell carcinoma	5	5
	poorly differentiated carcinoma	10	9
Tumor size			
	≤50mm	73	69
	50mm>	33	31
Number of tumor			
	single	93	88
	multi-tumor	13	12
Histology grade			0
	well and moderate	71	67
	poor	35	33
vascular invasion			0
	yes	9	8
	no	97	92
pulmonary tuberculosis			
	yes	3	3
	no	103	97
Pathology stage		0	
	I-II	79	75
	III-IV	27	25
Nodal status			
	p-N(0)	59	56
	p-N(1)-p-N(2)	47	44

Table S2. DNA sequences of real-time PCR primers used for the detection of mRNA expression.

primer name	Sequence (5’-3’)
ACTB Fwd	AGGCACCAGGGCGTGAT
ACTB Rev	GGGTGAGGATGCCTCTCTTG
AXIN-2 Fwd	CTTTCGCCAACCGTGGTT
AXIN-2 Rev	GGATCGCTCCTCTTGAAGGA
beta-catenin Fwd	TCCTGAGGAAGAGGATGTGGAT
beta-catenin Rev	CCTCTGAGCTCGAGTCATTGC
Claudin-1 Fwd	GAGCAGCACATTGCAAGCAA
Claudin-1 Rev	TCGTCTTCCAAGCACTTCATACA
c-myc Fwd	GAGCCCCTGGTGCTCCAT
c-myc Rev	GCCTGCCTCTTTTCCACAGA
Cyclin D1 Fwd	CTGGAGGTCTGCGAGGAACA
Cyclin D1 Rev	AGCTGCAGGCGGCTCTTT
E-Cadherin Fwd	CTTTGACGCCGAGAGCTACAC
E-Cadherin Rev	TTGTCGACCGGTGCAATCT
FOXP3 Fwd	CCTACCCACTGCTGGCAAAT
FOXP3 Rev	CCTGGCAGTGCTTGAGGAA
GAPDH Fwd	GCACCACCAACTGCTTAGCA
GAPDH Rev	TCTTCTGGGTGGCAGTGATG
HER 2 Fwd	TCACCTACCTGCCCACCAAT
HER 2 Rev	GGGACCTGCCTCACTTGGTT
LEF-1 Fwd	CCACGACAAGGCCAGAGAAC
LEF-1 Rev	GGCATCATTATGTACCCGGAAT
MMP2 Fwd	GTGGGACAAGAACCAGATCACA
MMP2 Rev	CACATCGCTCCAGACTTGGA
MMP7 Fwd	CGGGAGGCATGAGTGAGCTA
MMP7 Rev	TTGGCTTCTAAACTGTTGGCATT
MMP9 Fwd	CGCCAGTCCACCCTTGTG
MMP9 Rev	TGCCACCCGAGTGTAACCAT
N-Cadherin Fwd	TTGAGCCTGAAGCCAACCTT
N-Cadherin Rev	TGTAGGTGGCCACTGTGCTTAC
Slug Fwd	TCCTGGTCAAGAAGCATTTCAA
Slug Rev	TTGTGGTATGACAGGCATGGA
Snail Fwd	ATGCCGCGCTCTTTCCT
Snail Rev	CGTAGGGCTGCTGGAAGGTA
TWIST1 Fwd	CAGGTACATCGACTTCCTCTACCA
TWIST1 Rev	TCCATCCTCCAGACCGAGAA
TWIST2 Fwd	CCGCCAGGTACATAGACTTCCT
TWIST2 Rev	CACACGGAGAAGGCGTAGCT
VIM Fwd	TTGCAGGAGGAGATGCTTCA
VIM Rev	GATTCCACTTTGCGTTCAAGGT
ZEB1 Fwd	GAAAGGAAGGGCAAGAAATCCT
ZEB1 Rev	TCTGCATCTGACTCGCATTCA
ZEB2 Fwd	GTGCAAGAGGCGCAAACAA
ZEB2 Rev	CCGTCATCCTCAGCAATATGAA

Table S3. Clinical characteristics and distribution of patients according to their FOXP3 expression levels.

Characteristics	Tumor FOXP3 expression			
	low level	high level	χ^2	p
All	65	41		
Age	66.4±8.4	67.2±11.4	t=0.3918	0.696
Gender				
Male	46	29	0	0.997
Female	19	12		
Smoking status			0.824	0.364
smoker	36	19		
non-smoker	29	22		
Histology			1.31	0.73
squamous cell carcinoma	17	10		
adenocarcinoma	39	25		
large cell carcinoma	2	3		
poorly differentiated carcinoma	7	3		
Tumor size				
≤50mm	45	28	0.01	0.919
50mm>	20	13		
Number of tumor				
single	58	35	0.35	0.555
multi-tumor	7	6		
Histology grade			0.052	0.82
well and moderate	43	28		
poor	22	13		
vascular invasion			1.12	0.289
yes	7	2		
no	58	39		
pulmonary tuberculosis			0.037	0.847
yes	2	1		
no	63	40		
Pathology stage			1.369	0.242
I-II	51	28		
III-IV	14	13		
Nodal status			0.109	0.742
p-N(0)	37	22		
p-N(1)-p-N(2)	28	19		
Treg cell counts				
>25	19	24	8.95	0.003*
≤25	46	17		

Table S4. Cox proportional Hazard regression analysis of patients' overall survival.

Varibles	Univariable				Multivariable			
		95.0% CI				95.0% CI		
	HR	lower	upper	P	HR	lower	upper	P
Age	1.00	0.98	1.03	0.76	1.01	0.99	1.04	0.28
Gender (male vs female)	1.22	0.75	2.01	0.42	1.06	0.59	1.92	0.84
Smoking status (smoker vs nonsmoker)	1.10	0.71	1.72	0.67	0.88	0.52	1.48	0.63
Histology grade (poor vs well and moderate)	1.62	1.02	2.57	0.04*	2.10	1.29	3.41	0.00*
Tumor size (>50mm vs ≤50mm)	1.03	0.63	1.68	0.90	1.07	0.61	1.86	0.82
Pathology stage (III+IV vs I+II)	2.75	1.67	4.53	0.00*	1.91	0.92	3.96	0.08*
Tumor number (multi number vs single)	1.37	0.71	2.67	0.35	0.63	0.27	1.48	0.29
Vascular invasion (yes vs no)	1.21	0.56	2.64	0.63	1.10	0.46	2.66	0.83
Pathology TB (yes vs no)	2.96	0.91	9.60	0.07*	3.23	0.88	11.90	0.07*
Nodal status (pN(1)+pN(2) vs pN(0))	3.04	1.92	4.81	0.00*	2.96	1.68	5.18	0.00*
Treg cell counts >25 vs £25	1.05	0.67	1.65	0.84	0.95	0.57	1.61	0.86
FOXP3 expression (High vs Low)	1.86	1.18	2.92	0.01*	2.09	1.27	3.44	0.00*

Table S5. Cox proportional Hazard regression analysis of patients' recurrence-free survival.

Varibles	Univariable				Multivariable			
		95.0% CI				95.0% CI		
	HR	lower	upper	P	HR	lower	upper	P
Age	1.00	0.97	1.02	0.71	1.00	0.98	1.03	0.85
Gender (male vs female)	1.23	0.75	2.02	0.40	1.09	0.60	1.96	0.78
Smoking status (smoker vs nonsmoker)	1.11	0.71	1.73	0.64	0.84	0.50	1.42	0.52
Histology grade (poor vs well and moderate)	1.70	1.07	2.69	0.02*	1.97	1.20	3.24	0.01*
Tumor size (>50mm vs ≤50mm)	1.14	0.70	1.86	0.60	1.05	0.60	1.82	0.87
Pathology stage (III+IV vs I+II)	2.61	1.58	4.31	0.00*	1.66	0.82	3.36	0.16
Tumor number (multiple vs single)	1.61	0.83	3.15	0.16	0.73	0.31	1.69	0.47
Vascular invasion (yes vs no)	1.09	0.50	2.37	0.83	0.99	0.42	2.40	0.98
Pathology TB (yes vs no)	1.81	0.57	5.80	0.32	1.81	0.50	6.54	0.36
Nodal status (pN(1)+pN(2) vs pN(0))	2.59	1.64	4.09	0.00*	2.38	1.36	4.15	0.00*
Treg cell counts >25 vs ≤25	0.91	0.58	1.44	0.69	0.82	0.49	1.39	0.46
FOXP3 expression (High vs Low)	1.71	1.09	2.70	0.02*	1.99	1.15	3.46	0.01*
