# Supplementary material for: FOXP3 promotes tumor growth and metastasis by activating Wnt/β-catenin signaling pathway and EMT in non-small cell lung cancer
Source: Mol Cancer. 2017 Jul 17;16:124. doi: 10.1186/s12943-017-0700-1 (PMC5514503; doi:10.1186/s12943-017-0700-1)
Supplement: Supplementary file 2 — Supplementary Methods and Figures. (DOCX 3930 kb) [file 12943_2017_700_MOESM2_ESM.docx]

**Supplementary Methods and Figures**

**Supplementary Methods**

**Production of lentivirus for FOXP3 overexpression and knockdown**

The full-length ORF (open reading sequence) of human FOXP3 gene (NM_014009.3) was PCR amplified from pcDNA3.1-FOXP3 and subcloned into the self-inactivating lentiviral vector PHIV-EGFP (Addgene) with an EF1-alpha promoter, an internal ribosome entry site (IRES) and EGFP. Lentiviral preparations were generated by transient transfection of HEK-293T cells by using pHIV-EGFP-FOXP3 (10 µg), pRSV-Rev (2.2 µg), pMDLg/pRRE (4.72 µg), pMD2.G (3.08 µg)

FOXP3 shRNA was selected from the RNAi Consortium library (www.broadinstitute.org/rnai/public), which contains shRNAs against 15,000 human genes. We selected 5 highly scored target sequence against FOXP3 (NM_014009.3) mRNA sequence from the database: CCTCCACAACATGGACTACTT; CACACGCATGTTTGCCTTCTT; CTGAGTCTGCACAAGTGCTTT; TCCTACCCACTGCTGGCAAAT; TGTCCCTCACTCAACACAAAC. The corresponding oligoes generated by Invitrogen were subcloned into pLKO.1 (Addgene). Lentiviral preparations were generated as above except for that the transfection cocktail was replaced with pLKO.1 (6 µg), psPAX2 (4.5 µg) and pMD2.G (1.5 µg).

**Immunofluorescence assay**

The anti-E-Cadherin (Cellsignaling, 1:200) and anti-FOXP3 (Abcam, 1:100) were used as the primary antibodies. The cells were incubated with Alexa Fluor® 594 dye conjugated secondary antibody (ThermoFisher). The nucleus was stained by DAPI (ThermoFisher).

**Gene expression microarrays and data analysis**

Total RNA was extracted from A549-FOXP3 and A549-Control using TRIzol reagent (Invitrogen). The marked cRNAs were hybridized with the Agilent human whole genome gene expression Microarray (Agilent Technologies, Santa Clara, CA). Gene expression levels were standardized by the level of GAPDH. Differentially expressed genes were screened by the threshold of 2.0 fold-change and p value that was more than 0.05. Student’s t test was adopted for statistical analysis. Pathway analysis and Gene Ontology (GO) analysis were applied to determine the functions of those differentially expressed mRNAs by GO (www.geneontology.gov) and the KEGG (Kyto Encyclopedia of Genes and Genomes) pathway database (http://www.genome.jp/kegg/pathway.html).

**Supplementary Figures：**


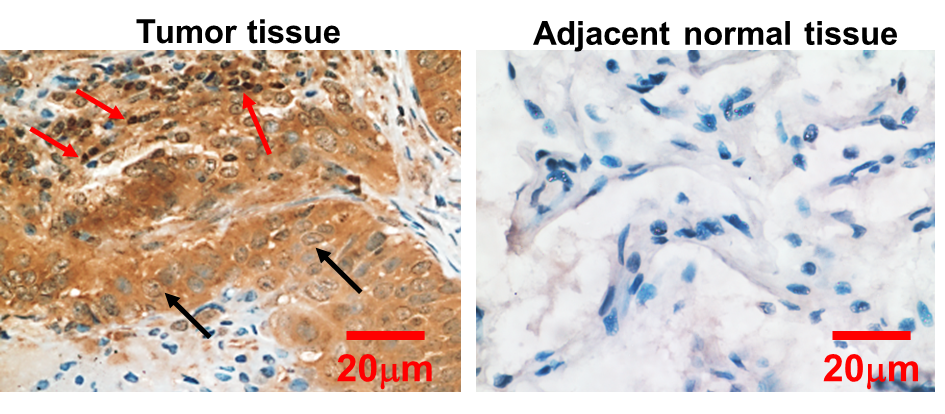


**Figure S1.** Immunohistochemical staining of FOXP3 in NSCLC tissues. FOXP3 is highly expressed in the nucleus and cytoplasm of NSCLC cells compared to adjacent normal tissue cells. (Red arrows: FOXP3^+^ Treg cells; black arrows: FOXP3^+^ tumor cells)


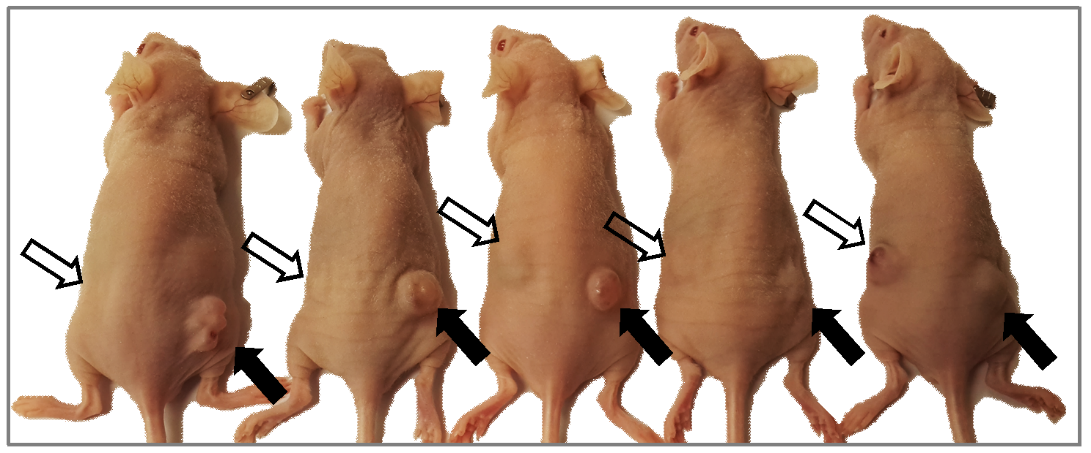

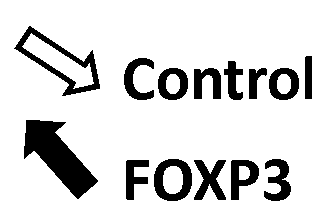


**Figure S2.** FOXP3 promotes NSCLC tumor growth in vivo. A549-Control cells were subcutaneously injected in left dorsal flank and A549-FOXP3 cells in right dorsal flank. Representative images of the tumors (arrows) in nude mice were taken 18 days after the injection.


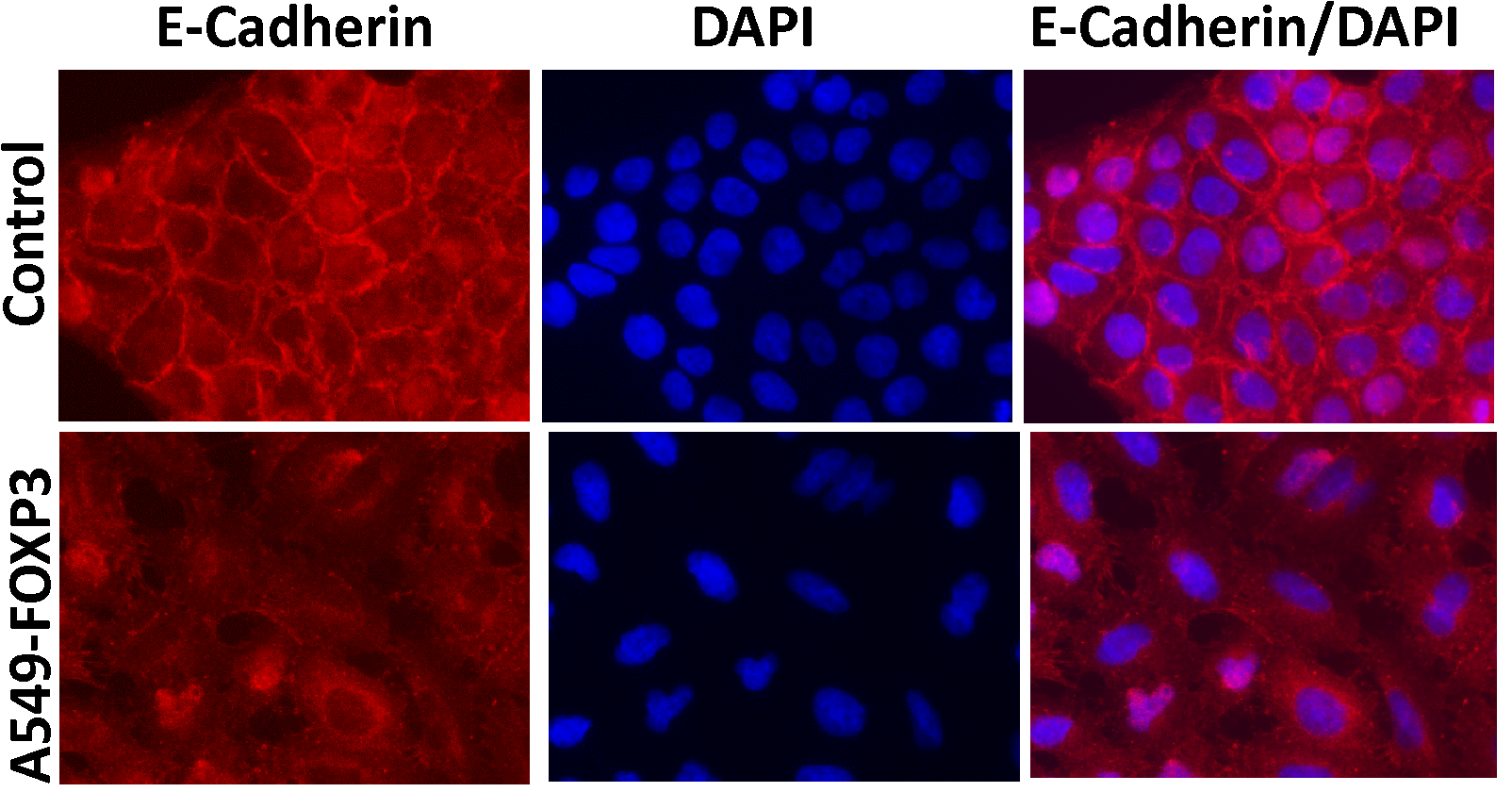


**Figure S3.** FOXP3 reduces the expression of E-Cadherin in NSCLC cells. E-Cadherin immunofluorescent staining demonstrated that E-Cadherin expression was markedly reduced in FOXP3-expressing A549 cells compared with control cells. Nuclei were counterstained with 40,6-diamidino-2-phenylindole (DAPI, blue).


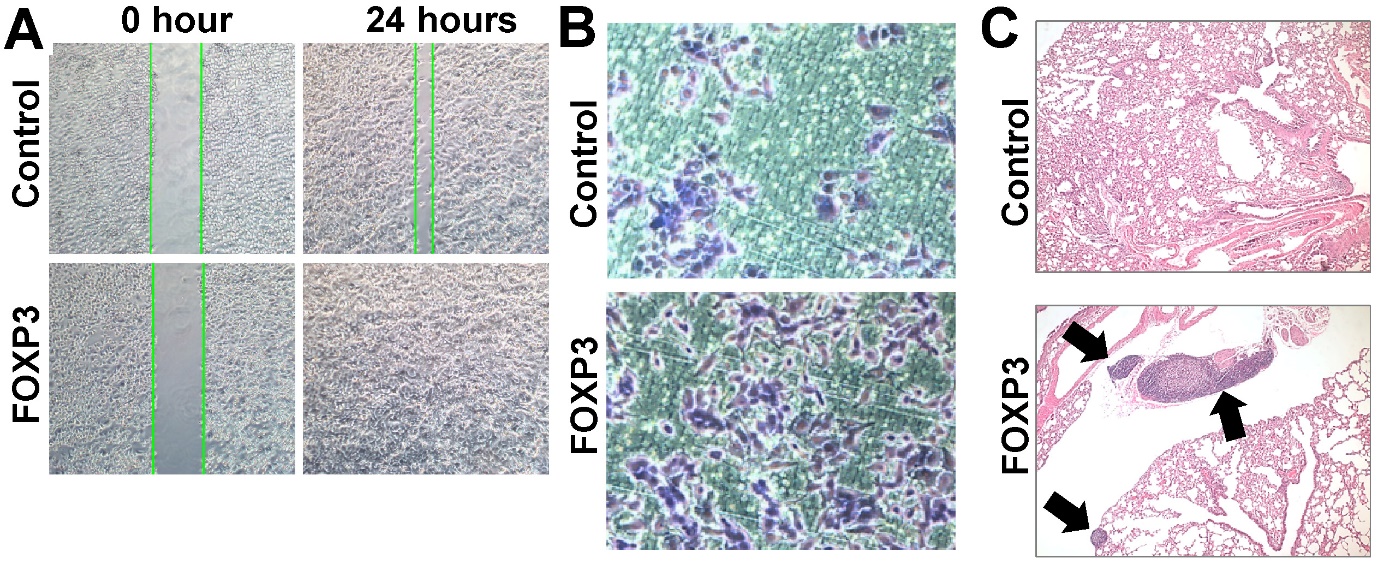


**Figure S4.** FOXP3 promotes tumor metastasis in NSCLC. (A) Wound healing assay showed that FOXP3-expressing A549 cells had a much stronger healing ability than control cells (p<0.01). (B) Matrigel invasion assay showed that FOXP3-expressing A549 cells had higher penetration rate through the Matrigel-coated membrane compared with control cells (p<0.001). (C) The total number of metastatic nodules, indicated by black arrow, was quantified in lungs of nude mice 8 weeks after tail vein injection of control and FOXP3-expressing A549 cells.


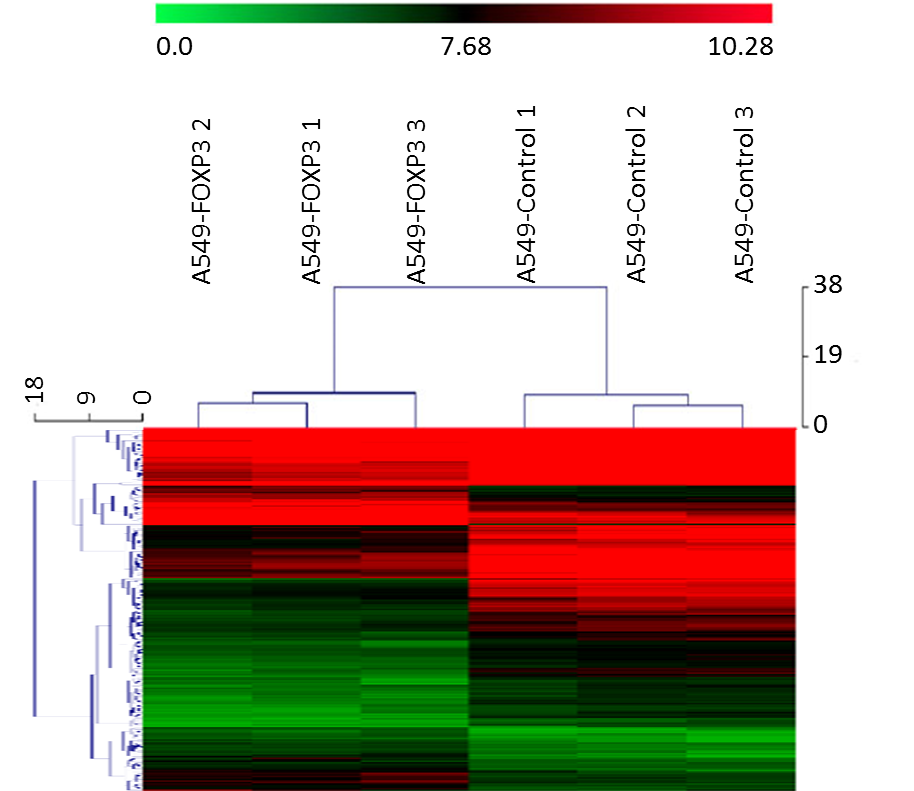


**Figure S5** (related to Figure 5). Hierarchical clustering of genes that were significantly and differentially expressed in A549-FOXP3 cells and control cells.


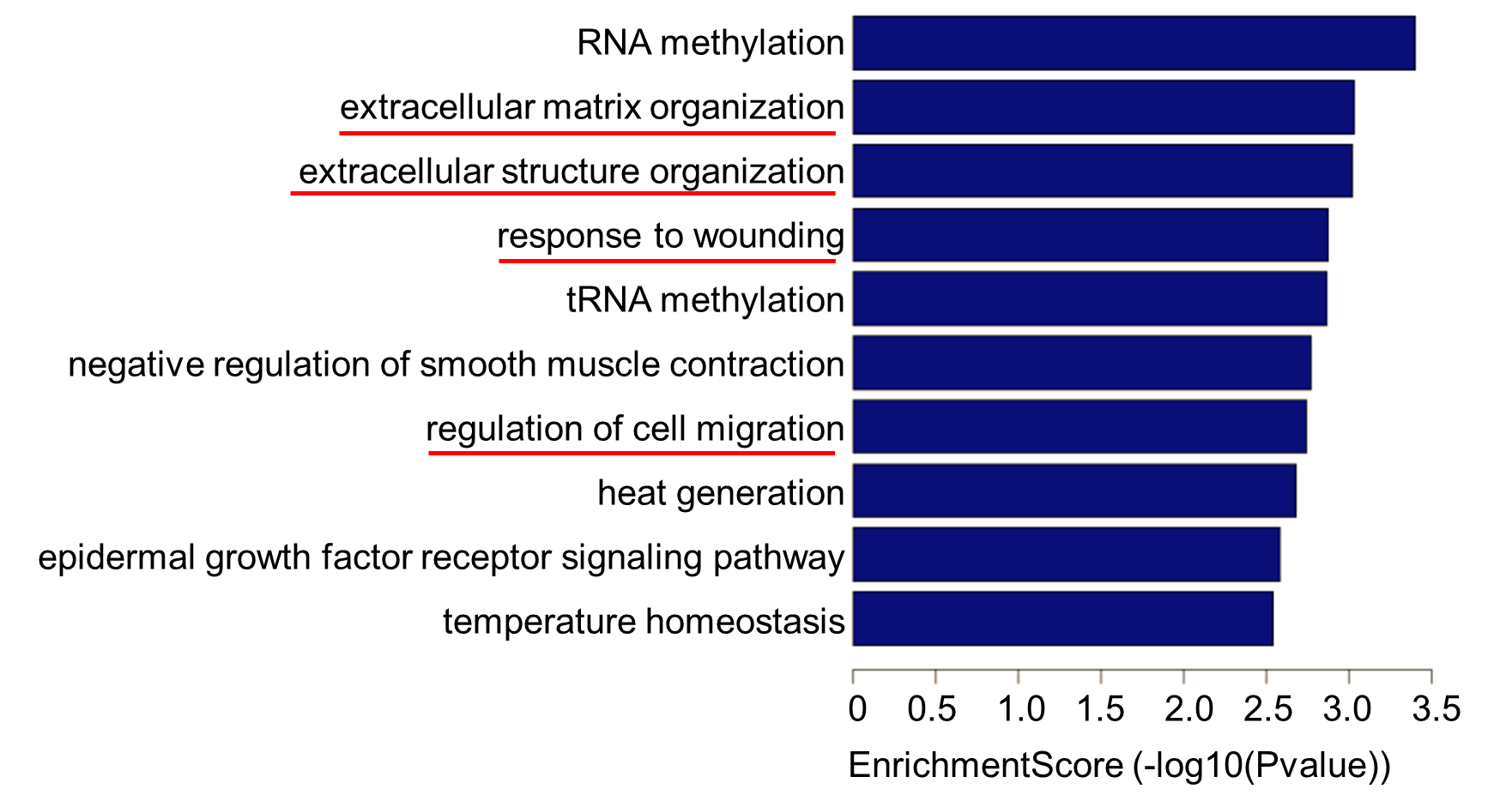


A

B


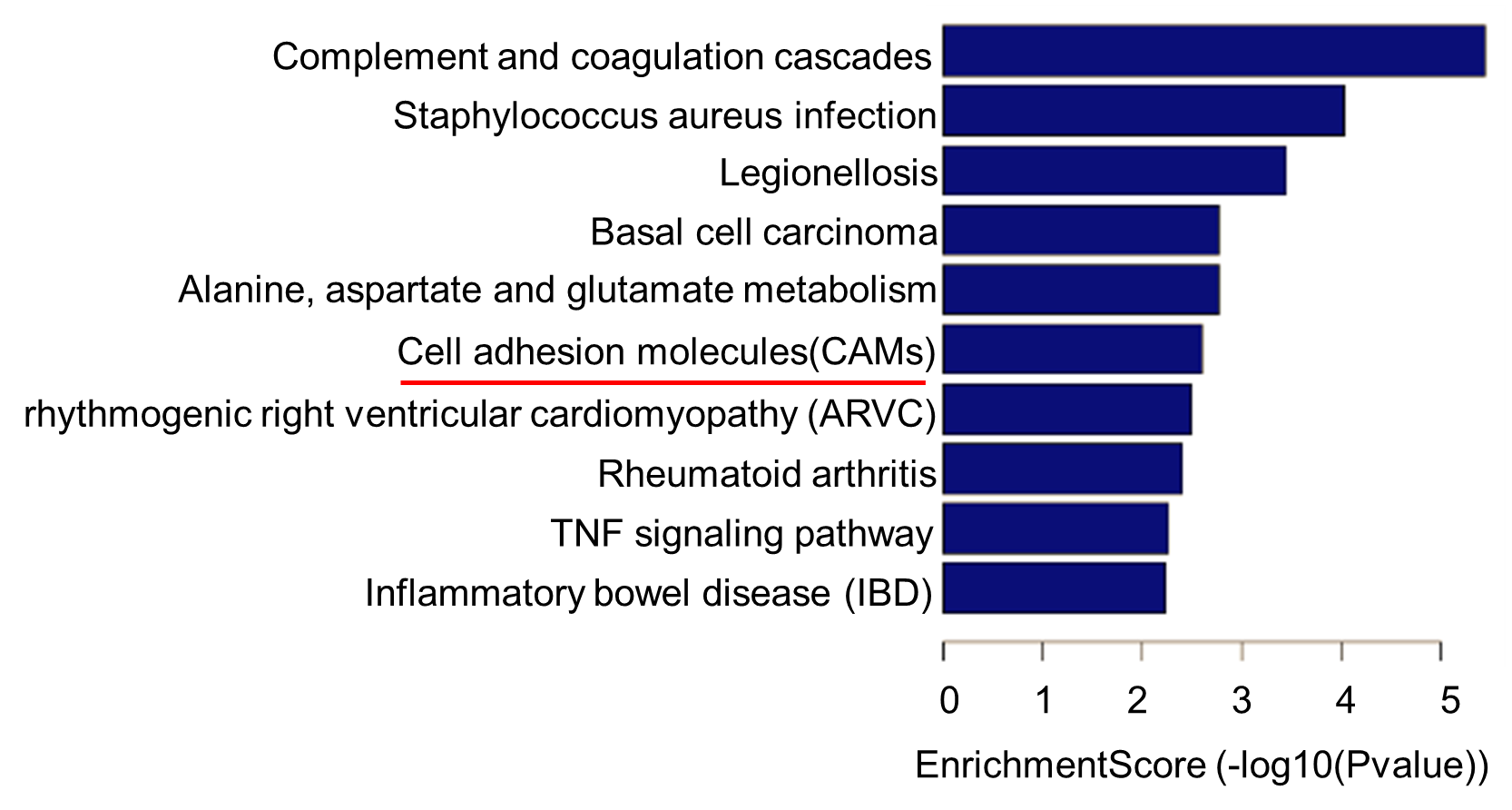


**Figure S6** (related to Figure 5). GO analysis Report based on microarray data (red line denotes relation to EMT): (A) Biological process. (B) Cellular Component


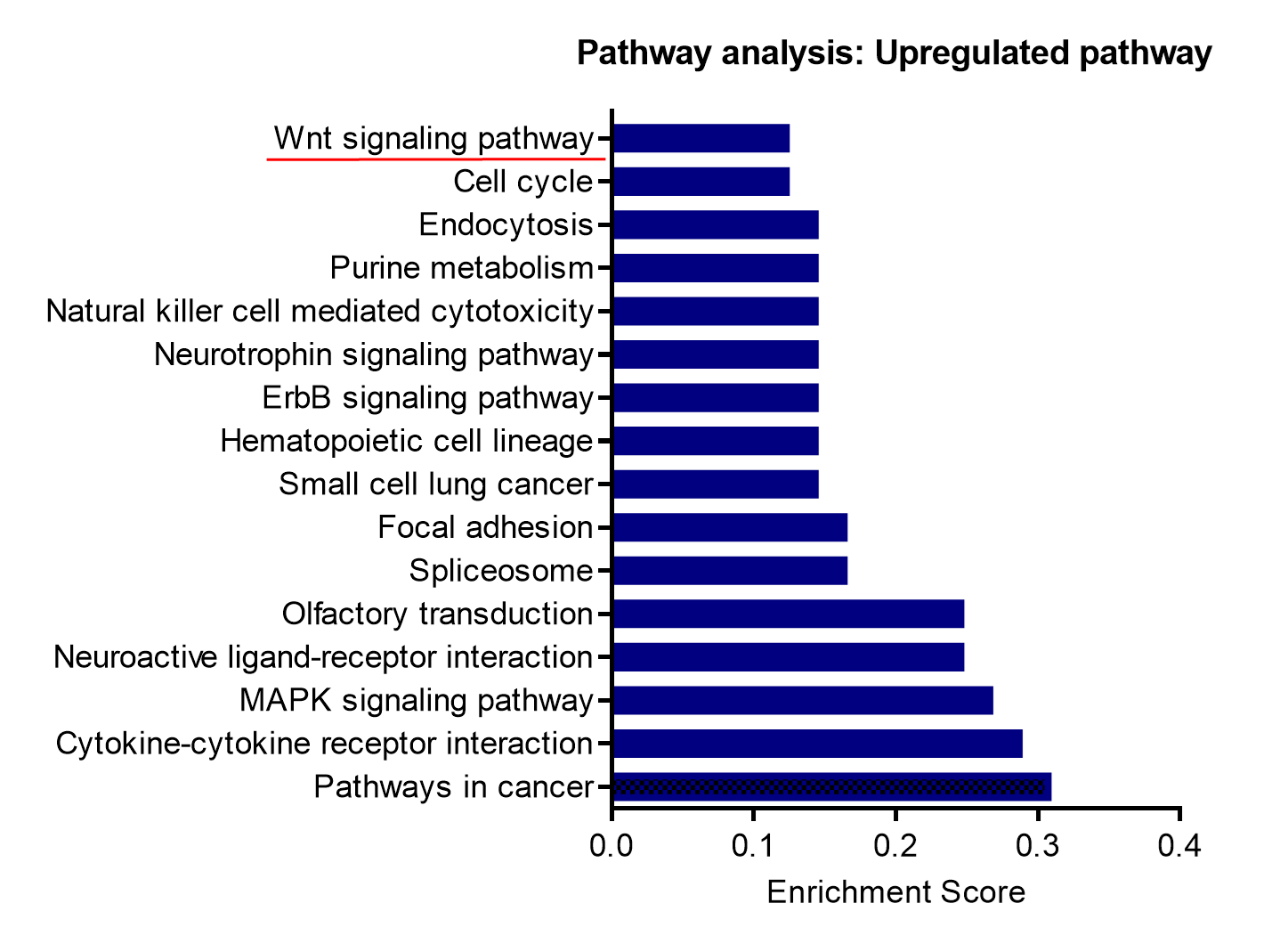
**Figure S7** (related to Figure 5). Pathway analysis of genes that were significantly and differentially upregulated in A549-FOXP3 cells and control cells using KEGG databa.


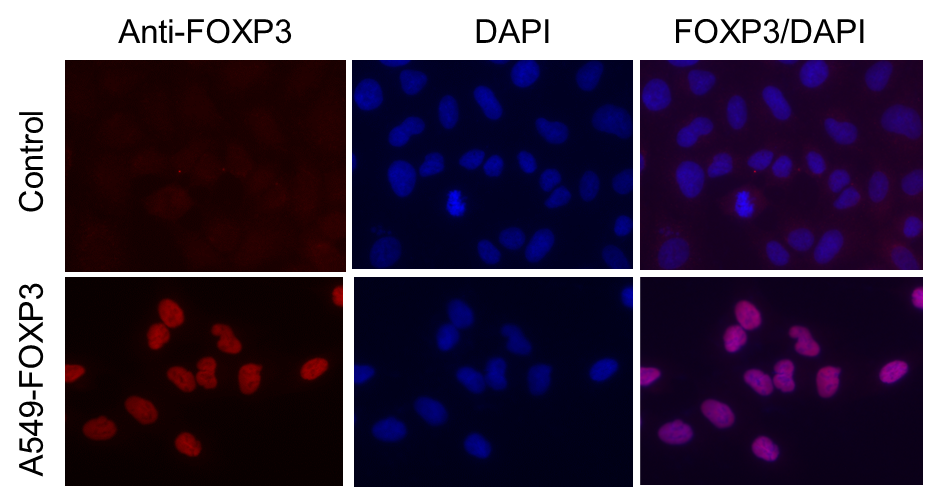


**Figure S8** (related to Figure 6). Immunofluorescent staining to detect FOXP3. FOXP3 immunofluorescent staining demonstrated that ectopic expression of FOXP3 was mainly in the nucleus.


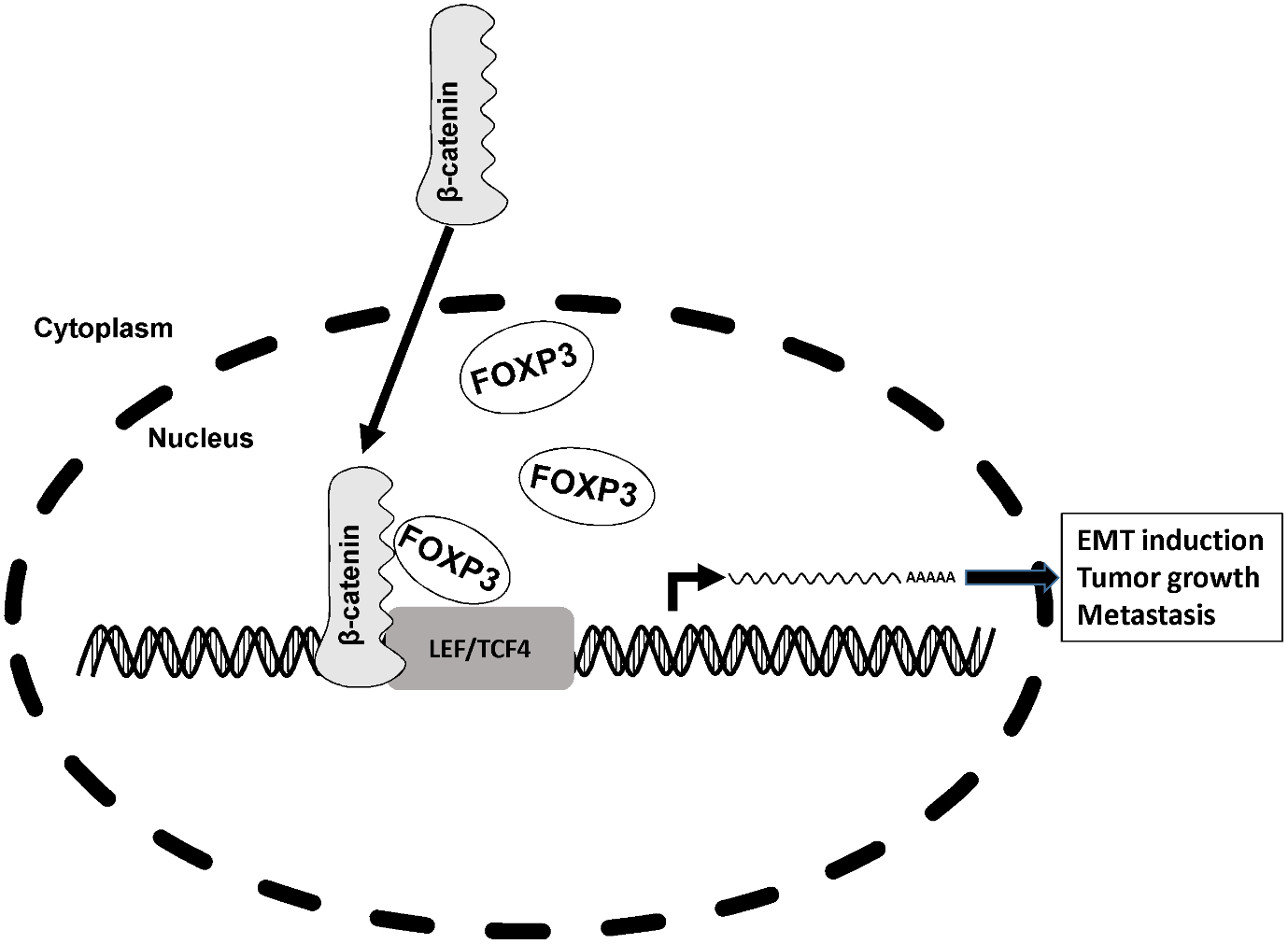


**Figure S9.** The proposed oncogenic mechanism of FOXP3 in NSCLC.
